# Schizophrenia-risk and urban birth are associated with proteomic changes in neonatal dried blood spots

**DOI:** 10.1038/s41398-017-0027-0

**Published:** 2017-12-18

**Authors:** Jason D. Cooper, Sureyya Ozcan, Renee M. Gardner, Nitin Rustogi, Susanne Wicks, Geertje F. van Rees, F. Markus Leweke, Christina Dalman, Håkan Karlsson, Sabine Bahn

**Affiliations:** 1 Department of Chemical Engineering and Biotechnology, University of Cambridge, Cambridge, UK; 2 Department of Public Health Sciences, Karolinska Institutet, Stockholm, Sweden; 3 Centre for Epidemiology and Community Medicine, Stockholm County Council, Stockholm, Sweden; 4 Brain and Mind Centre, University of Sydney, Sydney, Australia

## Abstract

In the present study, we tested whether there were proteomic differences in blood between schizophrenia patients after the initial onset of the disorder and controls; and whether those differences were also present at birth among neonates who later developed schizophrenia compared to those without a psychiatric admission. We used multiple reaction monitoring mass spectrometry to quantify 77 proteins (147 peptides) in serum samples from 60 first-onset drug-naive schizophrenia patients and 77 controls, and 96 proteins (152 peptides) in 892 newborn blood-spot (NBS) samples collected between 1975 and 1985. Both serum and NBS studies showed significant alterations in protein levels. Serum results revealed that Haptoglobin and Plasma protease C1 inhibitor were significantly upregulated in first-onset schizophrenia patients (corrected *P* < 0.05). Alpha-2-antiplasmin, Complement C4-A and Antithrombin-III were increased in first-onset schizophrenia patients (uncorrected *P-*values 0.041, 0.036 and 0.013, respectively) and also increased in newborn babies who later develop schizophrenia (*P-*values 0.0058, 0.013 and 0.044, respectively). We also tested whether protein abundance at birth was associated with exposure to an urban environment during pregnancy and found highly significant proteomic differences at birth between urban and rural environments. The prediction model for urbanicity had excellent predictive performance in both discovery (area under the receiver operating characteristic curve (AUC) = 0.90) and validation (AUC = 0.89) sample sets. We hope that future biomarker studies based on stored NBS samples will identify prognostic disease indicators and targets for preventive measures for neurodevelopmental conditions, particularly those with onset during early childhood, such as autism spectrum disorder.

## Introduction

Despite decades of research, the aetiology of schizophrenia is poorly understood. Schizophrenia is a severe and disabling psychiatric disorder involving impairments in perception, cognition and motivation that usually become evident in late adolescence or early adulthood. Early diagnosis of schizophrenia is beneficial for patients as shorter periods of untreated psychosis have been linked to better patient outcomes^1^. However, as there are no diagnostic tests for schizophrenia, diagnosis is still based on the evaluation of signs and symptoms in clinical interviews. Consequently, misdiagnosis is common^2^ as patients are required to acknowledge the occurrence of symptoms of psychosis, such as hallucinations and delusions. Furthermore, other psychiatric disorders can present with overlapping symptoms.

To date, most proteomic and biomarker studies have focused on the detection of changes in protein levels in patients with confirmed disease status versus healthy individuals. However, for certain adult onset diseases such as type 2 diabetes, hypertension and stroke, increasing attention is being given to detecting prognostic disease markers in early life and even in newborn babies^3^. Early detection of disease predisposition could allow for targeted prevention or amelioration of disease course before overt symptoms develop. This could be achieved by therapeutic or lifestyle interventions.

Since the late 1960s, newborn blood-spot (NBS) screening programs have become routine to test for rare but serious metabolic health conditions, such as cystic fibrosis and sickle cell disease. The stability of DNA, RNA, small molecules and proteins within the dried blood-spot (DBS), combined with the ease of collection, shipping and storage provide a powerful tool for screening programs and for large population-based surveys. DBS sampling will be particularly important for diseases like psychiatric disorders in which patient recruitment is notoriously difficult and expensive. We^4^ and other researchers^5^, have previously demonstrated the potential utility of DBS sampling for clinical proteomics and personalised medicine applications using multiple reaction monitoring (MRM). In MRM, a highly specific, reproducible and sensitive mass spectrometry (MS) technique, pre-defined protein peptides or small molecules of interest can be robustly quantified from small sample volumes.

Reported environmental risk factors for schizophrenia that potentially affect early neurodevelopment during pregnancy include infections^6^ and nutritional deficiencies^7^, intrauterine growth restriction^8^ and other pregnancy and birth complications. Established risk factors following birth include infections^6^, socioeconomic and childhood adversity. Epidemiological studies have also revealed an increased risk of developing psychiatric disorders for individuals born^9,10^ and living^11^ in urban environments. However, whether the effect of urbanicity on schizophrenia incidence is a consequence of unknown risk factors associated with place of birth, place of residence or both is unclear.

In the present study, we tested whether serum protein abundance differed between first-onset drug-naive schizophrenia patients and controls. We then tested whether those protein differences were present in NBS samples collected from newborn babies who later developed schizophrenia (‘future schizophrenia patients’) and those without a psychiatric admission. For the latter analysis, we had to initially determine whether we could detect the targeted protein peptides in stored NBS samples collected from neonates born in Sweden between 1975 and 1985. As our study population included babies exposed to urban and rural environments during pregnancy, we also tested whether protein abundance at birth was associated with urbanicity.

## Materials and methods

### Subjects

The Cologne study, as previously described^12,13^, consisted of serum samples from 60 first-onset drug-naive schizophrenia patients and 79 age and sex matched controls recruited by the Department of Psychiatry, University of Cologne (Table 1a). The ethical committees of the Medical Faculty of the University of Cologne and Addenbrooke’s Hospital (Cambridge, UK) approved the protocols of this study including procedures for sample collection and analysis. Informed consent was given in writing by all participants.

**Table 1:** Demographics of participants with proteomic data that survived the statistical quality control. (a) Cologne study: 60 first-onset drug naïve schizophrenia patients and 77 controls (two controls were excluded as outliers). (b) neonatal study: 172 psychosis patients, 75 schizophrenia patients and 644 controls

(a)					Cologne							
					Controls				Schizophrenia			
					M		F		M		F	
Number of subjects					43		34		31		29	
Age (years)					31.1 (8.6)		32.7 (9.0)		30.1 (10.2)		31.8 (10.8)	
Body mass index (kg/m^2^)					24.0 (3.2) (15 missing)		22.3 (3.7) (9 missing)		25.0 (5.7) (3 missing)		23.8 (5.4) (3 missing)	
Smoking					27.9% (12/43)		29.4% (10/34)		58.3% (14/24; 7 missing)		54.5% (12/22; 7 missing)	

(b)												
	Stockholm						Västerbotten and Norrbotten					
	Controls		Non-affective psychosis		Schizophrenia		Controls		Non-affective psychosis/affective psychosis		Schizophrenia	
	M	F	M	F	M	F	M	F	M	F	M	F
Number of subjects	239	223	64	56	24	13	92	90	14/5	20/13	25	13
Age of mother (years)	28.0 (5.1)	28.1 (4.8)	28.8 (5.7)	28.5 (5.3)	29.2 (7.1)	28.3 (5.5)	27.1 (5.1)	27.6 (5.5)	25.3 (5.9)	27.6 (5.5)	27.8 (3.8)	28.9 (5.1)
Completed weeks of gestation	39.6 (1.8)	39.8 (1.8)	39.1 (2.1)	39.7 (1.8)	39.4 (1.4)	39.8 (1.0)	39.4 (1.6)	39.4 (2.1)	39.4 (1.8)	39.4 (2.1)	39.5 (1.8)	39.7 (1.5)
Caesarean section	12.6%	14.8%	4.7%	5.4%	25.0%	0.0%	16.3%	6.7%	5.3%	21.2%	16.0%	7.7%
Head circumference (cm)	34.8 (1.6)	34.5 (1.7)	34.8 (1.6)	34.5 (1.6)	34.2 (2.0)	33.9 (1.6)	34.9 (1.7)	34.6 (1.9)	34.1 (1.5)	34.5 (1.9)	35.3 (1.6)	33.9 (1.8)
Birth weight (g)	3509(542)	3469(575)	3433(606)	3483(549)	3303(611)	3387(484)	3582(555)	3391(707)	3463(569)	3391(707)	3594(525)	3196(480)
Birth length (cm)	50.5 (2.3)	50.0 (2.3)	50.5 (2.6)	50.2 (2.1)	49.5 (2.5)	50.2 (1.7)	51.1 (2.3)	49.9 (3.0)	51.1 (2.3)	49.9 (3.0)	51.1 (2.2)	48.9 (2.3)

*F* female, *M* male. Values are presented as average (standard deviation)

The Stockholm population, as previously described^14^, consisted of all persons (born in Sweden 1975–1985) and treated for non-affective psychosis within psychiatric services in Stockholm County as inpatients (from 1987) or outpatients (from 1997 until 2004). The other population consisted of persons born between 1975 and 1985 in two Northern counties (Västerbotten and Norrbotten) and treated for non-affective and other psychoses between 1987 and 2005. Control subjects had no history of inpatient psychiatric admission, according to the National Patient Register^15^, and had to be alive and resident in Sweden. The controls were matched for sex, birth year and birth hospital. The aim was to recruit two controls per patient. Schizophrenia was defined as ICD9-code 295 (excluding 295F and 295H) or ICD10-code F20. Non-affective psychosis (excluding schizophrenia) was defined as ICD9-code 297, 298C–298X, 295F and 295H or ICD10-code F21–F29. The Northern Sweden data also included affective psychosis patients defined as ICD9-code 296 and 299 or ICD10-code F39, F333, F323, F315, F312 and F302. We only formed a ‘psychosis patient group’ consisting of patients with either non-affective or affective psychosis as a validation sample set to assess whether the urban–rural associations identified in controls could also be detected in an independent cohort (i.e. the future disease status is not relevant for this comparison). At the end of December 2003, the neonatal study consisted of 645 controls (no psychiatric diagnosis, subsequently referred to as ‘controls’), 172 psychosis patients and 75 schizophrenia patients (Table 1b). All samples were stored in the same NBS sample repository in Stockholm. We obtained the following information through linkage to the Medical Birth Register:^16^ gestational age at birth, birth weight and length, birth order, Apgar score, head circumference, maternal eclampsia, maternal immigration, maternal age and place of residency (municipality) at delivery. Data on population density (number of inhabitants per km^2^) for each municipality in 1991 was obtained from Statistics Sweden. The study was approved by the regional ethics committee in Stockholm and all participants provided their signed consent.

### Targeted protein quantification in NBS and serum

NBS and serum samples were prepared in a 96 plate format as described previously^4^. Briefly, proteins were extracted from serum and NBS samples using ammonium bicarbonate. Then, disulphide bond reduction and cysteine alkylation were performed using dithiothreitol and iodoacetamide, respectively. Proteins digested overnight using trypsin (Supplementary Information). Isotopically labelled internal standard peptides were spiked into both NBS and serum samples prior to MS run. Quality control (QC) samples were used in this study to monitor method performance and instrument stability (Supplementary Information).

In this study, a total of 101 serum proteins (172 peptides), the majority previously associated with psychiatric disorders, were selected. Three to four interference-free transitions were selected for each targeted peptide as described previously^4^. Tryptic digested peptides were monitored using an Agilent 1290 Liquid Chromatography (LC) system coupled with 6495 Triple Quadrupole MS equipped with jet-stream nano ESI source operated in positive mode. MS data were acquired in MRM mode. The chromatographic separation was carried out on Agilent AdvanceBio Peptide Map column (2.1 × 150 mm 2.7-micron) at 50 °C. Peptides were eluted over a linear gradient from 3% to 30% acetonitrile in 0.1% formic acid in 45 min.

### Statistical analysis

#### Data pre-processing and quality control

Raw MS files were processed using the Skyline software package (Version 3.1.0). Peaks were manually checked, and peak integrations were adjusted accordingly when necessary. The endogenous and internal standard peptide-transition peak areas were estimated and exported as a comma delimited data file for statistical analysis in R (Version 3.2.3)^17^. MS data pre-processing is described in the Supplementary Information.

#### Coefficient of variation

We used the geometric coefficient of variation (CV), which describes the amount of variability relative to the mean, to quantify the degree of variation for the peptides across the MS runs. For natural log transformed data, the geometric CV = $\sqrt {e^{{\mathrm{sd}}^2} - 1} \times 100$ (ref. 18), where sd is the standard deviation of the log-transformed data. Note that the geometric CV was used as it is important to estimate the variability on the original scale of measurement.

#### Patient-control association analysis

We tested the association between relative peptide abundance and disease status (0 control and 1 schizophrenia) in the Cologne study using a logistic regression model. As body mass index was missing for over 20% of the participants and smoking for over 20% of patients (Table 1a), only age and sex were available for selection. In the analysis of schizophrenia patients and controls from the neonatal study, we used a generalised additive model (GAM)^19^. As proteins dried on filter paper can degrade overtime^20^ and degradation may not be a linear function of time, we used a GAM to allow for a smooth to be fitted for year of birth, which represents the time of storage. The smooth may also better fit any changes in protein decay associated with the 1981 change in the storage of the Swedish NBS collection cards from room temperature to 4° and 30% humidity. In the R package mgcv^21^, smooth functions of the GAM are represented using penalised regression splines. The following covariates were available for selection: sex, year of birth (linear or smooth; Supplementary Table 1), whether the mother was born abroad, Apgar score at 1 min, Apgar score at 5 min, parity, whether the child was the first born, caesarean section, completed weeks of gestation, birth weight, length at birth, head circumference, whether the baby was small for their gestation age, age of mother, whether the mother suffered from eclampsia, and population density of the municipality where the mother was living at the time of the birth of the child (grouped as 0.1–49, 50–99, 100–499, 500–999, 1000–2999 and 3000–3999 per km^2^). We used the R package mice^22^ to replace missing covariate values using multiple-imputation (Supplementary Table 2). Model selection was based on forward-selection with Bayesian information criterion. We also fitted a joint effects model to predict disease status using ten-fold cross-validation with least absolute shrinkage and selection operator (lasso; Supplementary Information) regression as implemented in the R package glmnet^23,24^.

#### Urban–rural association analysis

We tested the association between relative peptide abundance and urbanicity at birth (0 rural and 1 urban) in controls from the neonatal study using a GAM. Rural was defined as a population density <50 per km^2^ and urban centre as a population density ≥1000 per km^2^ (1500 per km^2^ used by European Union Organisation for Economic Co-operation and Development^25^ which falls within our population density group 1000–2999 per km^2^), respectively containing 182 and 214 controls. Model selection, including lasso regression, and variables available for selection were as in the case–control comparison. We attempted to validate the urbanicity prediction model in 34 rural and 45 urban future (affective and non-affective) psychosis patients from the neonatal study. However, only location of birth and not future disease status were relevant for this comparison.

We measured the predictive performance of the diagnostic biomarker panel using sensitivity, specificity and area under the receiver operating characteristic curves (AUC: 0.9–1 = excellent; 0.8–0.9 = good; 0.7–0.8 = fair; 0.6–0.7 = poor; 0.5–0.6 = fail). Optimal trade-offs between sensitivity and specificity were determined by maximising Youden’s index *J;* where *J* = sensitivity + specificity − 1.

## Results

### Targeted protein detection and their coefficients of variation

We monitored 77 proteins (147 peptides) in 139 serum samples from Cologne. These samples were randomly assigned to two 96-well plates, the second plate was half filled, and run over one and a half weeks on the MS. We used the CV to quantify the degree of variation (robustness) in the relative peptide abundances measured in a pooled serum sample. The median CV across the plates was 7.23% (6.54% in plate 1 and 7.92% in plate 2; Supplementary Fig. 1a).

As we have previously only processed DBS samples within 6 months of collection^4^, we had to determine whether we could detect the targeted protein peptides in stored NBS samples collected between 1975 and 1985 (Supplementary Table 1). We initially tested ten samples collected in 1975 and 1985, five from each year. A total of 101 serum proteins were monitored in these test samples (data not shown) and 96 proteins (152 peptides) were selected and subsequently, monitored in 892 NBS samples. The samples were randomly assigned to ten 96-well plates and run over 10 weeks on the MS.

The median CV for the relative peptide abundances measured in a pooled NBS sample across plates 2–10 was 10.83% (range 9.50–11.52%; Supplementary Fig. 1b), clearly demonstrating that we could reproducibly measure the targeted peptides in stored NBS samples collected between 1975 and 1985.

### Patient and control analysis

**Fig. 1:**
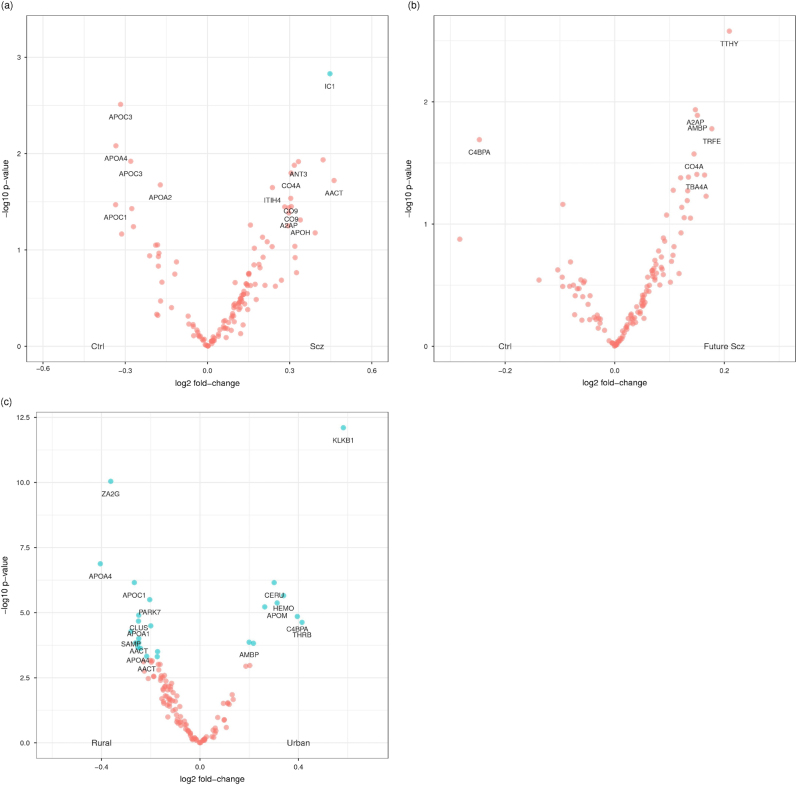
**a** A volcano plot summarising the association between protein abundance in 60 first-onset schizophrenia patients and 77 controls from Cologne (Table 2a). Light blue points indicate proteins that were significant after correction for multiple testing using the false discovery rate and had a fold-change >10%. Labelled proteins had uncorrected *P *< 0.05. **b** A volcano plot summarising the association between protein abundance in 75 future schizophrenia patients and 644 controls from the neonatal study (Table 2c). Note that none of the protein changes remained significant after correction for multiple testing using the false discovery rate. Labelled proteins had uncorrected *P* < 0.05. Interestingly, we identified a significantly greater number of increased proteins in the blood of newborn babies who were later diagnosed with schizophrenia. **c** A volcano plot summarising the association between protein abundance in urban and rural environments at birth. Note that serum albumin (ALBU) was excluded for display purposes only (Table 3). Light blue points indicate proteins that were significant after correction for multiple testing using the false discovery rate and had a fold-change >10%. Ctrl control, Scz schizophrenia

After QC, we analysed 68 proteins (128 peptides; Supplementary Table 3) in 60 first-onset drug-naive schizophrenia patients and 77 controls from Cologne. A total of 14 proteins (22 peptides) had an uncorrected *P* < 0.05 for abundance differences between patients and controls (Table 2a). After *P*-values were corrected for multiple testing, three Haptoglobin (HPT) peptides and a Plasma Protease C1 Inhibitor (IC1) peptide were significant. The volcano plot suggested that there were more peptide-transitions with higher abundances in schizophrenia patients than would be expected by chance alone (Fig. 1a); which would result in a more symmetric pattern about around the log_2_ fold-change of zero. We note that only the apolipoproteins A2, A3, A4, C1 and C3 were downregulated in patients compared to controls (Table 2a; Fig. 1a). In total, 13 of these 14 proteins have previously been associated with schizophrenia (Table 4). Although IC1 has not been linked to schizophrenia before, recent reports have linked IC1 dysregulation to Alzheimer’s disease^26,27^. The lasso prediction model consisted of 11 proteins (11 peptides; Supplementary Table 6a), and despite the absence of a clinical rating scale predictor, had a good predictive performance (area under the receiver operating characteristic curve (AUC = 0.80)).

**Table 2:** (a) The most associated protein peptides with an uncorrected *P* < 0.05 for the difference between 60 first-onset schizophrenia patients and 77 controls from the Cologne study. (b) The association for 12 of the 14 proteins reported in (a) and available in the 75 future schizophrenia patients and 644 controls from the neonatal study. (c) The most associated protein peptides with an uncorrected *P* < 0.10 for the 75 future schizophrenia patients and 644 controls from the neonatal study

(a) Cologne study				
Protein	Peptide	First-onset schizophrenia and controls		
		Fold-change	*P*	Corrected *P*
Haptoglobin (HPT)	VTSIQDWVQK	1.54	0.000535	0.0283
Haptoglobin (HPT)	DYAEVGR	1.56	0.000542	0.0283
Haptoglobin (HPT)	VGYVSGWGR	1.53	0.000653	0.0283
Plasma protease C1 inhibitor (IC1)	TNLESILSYPK	1.36	0.001480	0.0481
Apolipoprotein C-III (APOC3)	GWVTDGFSSLK	−1.25	0.00308	0.0801
Apolipoprotein A-IV (APOA4)	IDQNVEELK	−1.26	0.00829	0.172
Plasma protease C1 inhibitor (IC1)	FQPTLLTLPR	1.34	0.0116	0.172
Apolipoprotein C-III (APOC3)	DALSSVQESQVAQQAR	−1.21	0.0120	0.172
Antithrombin-III (ANT3)	FDTISEK	1.26	0.0121	0.172
Antithrombin-III (ANT3)	LPGIVAEGR	1.25	0.0132	0.172
Complement C4-A (CO4A)	VLSLAQEQVGGSPEK	1.24	0.0159	0.188
Alpha-1-antichymotrypsin (AACT)	EQLSLLDR	1.38	0.0190	0.206
Apolipoprotein A-II (APOA2)	SPELQAEAK	−1.13	0.0211	0.209
Inter-alpha-trypsin inhibitor heavy chain H4 (ITIH4)	GPDVLTATVSGK	1.18	0.0225	0.209
Complement component C9 (CO9)	VVEESELAR	1.23	0.0291	0.242
Apolipoprotein C-I (APOC1)	EFGNTLEDK	−1.26	0.0339	0.242
Complement component C9 (CO9)	LSPIYNLVPVK	1.24	0.0354	0.242
Complement C4-A (CO4A)	ITQVLHFTK	1.22	0.0357	0.242
Ficolin-3 (FCN3)	YGIDWASGR	1.23	0.0364	0.242
Apolipoprotein A-IV (APOA4)	ISASAEELR	−1.21	0.0372	0.242
Alpha-2-antiplasmin (A2AP)	FDPSLTQR	1.23	0.0412	0.255
Beta-2-glycoprotein 1 (APOH)	EHSSLAFWK	1.26	0.0488	0.288

(b) Neonatal study				
Protein	Peptide	Future schizophrenia and controls		
		Fold-change	*P*	^a^One-sided *P*
Haptoglobin (HPT)	VTSIQDWVQK	—	—	—
Haptoglobin (HPT)	DYAEVGR	—	—	—
Haptoglobin (HPT)	VGYVSGWGR	—	—	—
Plasma protease C1 inhibitor (IC1)	TNLESILSYPK	1.001	0.9710	—
Apolipoprotein C-III (APOC3)	GWVTDGFSSLK	1.002	0.9750	—
Apolipoprotein A-IV (APOA4)	IDQNVEELK	1.123	0.0592	^b^
Plasma protease C1 inhibitor (IC1)	FQPTLLTLPR	—	—	—
^c^Apolipoprotein C-III (APOC3)	DALSSVQESQVAQQAR	1.005	0.9200	—
Antithrombin-III (ANT3)	FDTISEK	—	—	—
Antithrombin-III (ANT3)	LPGIVAEGR	1.092	0.0887	0.0444
Complement C4-A (CO4A)	VLSLAQEQVGGSPEK	1.061	0.1860	—
^c^Alpha-1-antichymotrypsin (AACT)	EQLSLLDR	1.062	0.2340	—
^c^Apolipoprotein A-II (APOA2)	SPELQAEAK	1.120	0.0397	^b^
Inter-alpha-trypsin inhibitor heavy chain H4 (ITIH4)	GPDVLTATVSGK	1.015	0.7250	—
Complement component C9 (CO9)	VVEESELAR	—	—	—
Apolipoprotein C-I (APOC1)	EFGNTLEDK	1.030	0.5260	—
^c^Complement component C9 (CO9)	LSPIYNLVPVK	1.003	0.9700	—
^c^Complement C4-A (CO4A)	ITQVLHFTK	1.105	0.0267	0.01340
Ficolin-3 (FCN3)	YGIDWASGR	—	—	—
Apolipoprotein A-IV (APOA4)	ISASAEELR	1.087	0.1180	—
Alpha-2-antiplasmin (A2AP)	FDPSLTQR	1.107	0.0116	0.00580
Beta-2-glycoprotein 1 (APOH)	EHSSLAFWK	1.073	0.2990	—

(c) Neonatal study				
Protein	Peptide	Future schizophrenia and controls		
		Fold-change	*P*	
Transthyretin (TTHY)	VLDAVR	1.156	0.00264	
Alpha-2-antiplasmin (A2AP)	FDPSLTQR	1.1074	0.0116	
Protein AMBP (AMBP)	ETLLQDFR	1.1102	0.0129	
Serotransferrin (TRFE)	EGYYGYTGAFR	1.1307	0.0166	
C4b-binding protein alpha chain (C4BPA)	YTCLPGYVR	−1.1866	0.0204	
Complement C4-A (CO4A)	ITQVLHFTK	1.1055	0.0267	
Tubulin alpha-4A chain (TBA4A)	EIIDPVLDR	1.1094	0.0392	
Apolipoprotein A-II (APOA2)	SPELQAEAK	1.1203	0.0397	
Clusterin (CLUS)	IDSLLENDR	1.0977	0.0413	
Ig gamma-3 chain C region (IGHG3)	DTLMISR	1.0869	0.0418	
Kininogen-1 (KNG1)	DFVQPPTK	1.0769	0.0529	
Ig gamma-3 chain C region (IGHG3)	NQVSLTCLVK	1.0967	0.0534	
Apolipoprotein A-IV (APOA4)	IDQNVEELK	1.1225	0.0592	
Apolipoprotein D (APOD)	VLNQELR	1.0961	0.0643	
Purine nucleoside phosphorylase (PNPH)	VFGFSLITNK	−1.0679	0.069	
Apolipoprotein A-I (APOA1)	ATEHLSTLSEK	1.0885	0.073	
Alpha-2-antiplasmin (A2AP)	DFLQSLK	1.0677	0.0844	
Antithrombin-III (ANT3)	LPGIVAEGR	1.0918	0.0887	
Histone H4 (H4)	DAVTYTEHAK	1.1005	0.0894	

*P*-values were corrected for multiple testing using the false discovery rate. The selected covariates are listed in Supplementary Table 4a. The table also includes APOA4 (IDQNVEELK), ANT3 (LPGIVAEGR), APOA2 (SPELQAEK), CO4A (ITQVLHFTK) and A2P2 (FDPSLTQR) from (b). The selected covariates are listed in Supplementary Table 4b. ^a^One-sided test conducted when the direction of the fold-change is consistent with that from the Cologne study and the two-sided *P* < 0.10. ^b^Two-sided *P* < 0.10, but direction of the fold-change is not consistent. ^c^Same protein peptide but different transition in (b) compared to (a)

We then investigated whether any of these 14 proteins (Table 2a) also differed in abundance at birth. To this end, we analysed NBS samples obtained from 75 future schizophrenia patients and 644 controls. In total, 12 of the 14 proteins were available for analysis. We found Alpha-2-antiplasmin (A2AP), Complement C4-A (CO4A) and Antithrombin-III (ANT3) to be significantly different at birth (one-sided *P* < 0.05; Table 2b) as well as after the onset of the disorder. We also analysed the remaining 73 proteins (110 peptides) measured in the neonatal study to investigate whether the abundance of any other proteins was significantly different at birth. No other proteins were significantly different between future schizophrenia patients and controls after *P*-values were corrected for multiple testing (Table 2c). As in the first-onset schizophrenia analysis (Fig. 1a), there were more peptide-transitions with higher abundances in future schizophrenia patients compared to controls (Fig. 1b).

### Urbanicity

As birth in an urban environment has been associated with an increased risk for psychiatric disorders, we analysed 85 proteins (125 peptides) measured in 396 controls, 214 from urban and 182 from rural environments, to test whether protein abundance ratios at birth differed by urban–rural environment. Abundancies of 24 proteins (26 peptides) differed significantly after *P*-values were corrected for multiple testing and had a fold-change >10% (Fig. 1c; Table 3). We then attempted to validate these 24 proteins using NBS from 79 psychosis patients, 45 from urban and 34 from rural environments. Fifteen of the 24 proteins were validated (16 peptides; one-sided *P* < 0.05; Table 3). We did not attempt to further validate the associations in the future schizophrenia patients because of the relatively small number of patients from urban and rural environments, 17 and 38 respectively.

**Table 3:** Peptide-transitions significantly associated with urban–rural environment at birth

Protein	Peptide	Controls			Psychosis	
		Fold-change	*P*	Corrected *P*	Fold-change	One-sided P
Serum albumin (ALBU)	ETYGEMADCCAK	3.220	3.79E−29	3.15E−27	4.050	7.29E−10
Plasma kallikrein (KLKB1)	LSMDGSPTR	1.497	7.85E−13	6.52E−11	1.818	1.19E−06
Zinc-alpha-2-glycoprotein (ZA2G)	AGEVQEPELR	−1.286	9.00E−11	7.47E−09	−1.211	0.0117
Apolipoprotein A-IV (APOA4)	IDQNVEELK	−1.324	1.32E−07	1.10E−05	−1.254	0.0143
Apolipoprotein C-I (APOC1)	EFGNTLEDK	−1.203	6.98E−07	5.79E−05	−1.216	0.0147
Ceruloplasmin (CERU)	EVGPTNADPVCLAK	1.232	7.00E−07	5.81E−05	1.222	0.0129
Hemopexin (HEMO)	VDGALCMEK	1.265	2.21E−06	0.00018343	1.086	0.268
Protein deglycase DJ-1 (PARK7)	DGLILTSR	−1.152	3.16E−06	0.00026228	−1.135	0.0345
Apolipoprotein M (APOM)	SLTSCLDSK	1.243	4.23E−06	0.00035109	1.356	0.000394
Fructose-bisphosphate aldolase A (ALDOA)	ALANSLACQGK	1.200	5.98E−06	0.00049634	1.271	0.00479
Clusterin (CLUS)	IDSLLENDR	−1.188	1.25E−05	0.0010375	−1.233	0.0122
C4b-binding protein alpha chain (C4BPA)	YTCLPGYVR	1.315	1.40E−05	0.001162	−1.015	^a^
Apolipoprotein A-I (APOA1)	ATEHLSTLSEK	−1.189	2.11E−05	0.0017513	−1.009	0.462
Prothrombin (THRB)	SGIECQLWR	1.333	2.35E−05	0.0019505	1.356	0.00339
Apolipoprotein E (APOE)	AATVGSLAGQPLQER	−1.148	3.20E−05	0.002656	−1.019	0.385
Serum amyloid P-component (SAMP)	IVLGQEQDSYGGK	−1.214	5.24E−05	0.0043492	−1.161	0.0623
Alpha-1-antichymotrypsin (AACT)	EQLSLLDR	−1.188	9.81E−05	0.0081423	−1.237	0.0104
Protein AMBP (AMBP)	TVAACNLPIVR	1.148	0.000136	0.011288	1.118	0.0581
Protein S100-A6 (S10A6)	LQDAEIAR	−1.188	0.000144	0.011952	−1.015	0.438
Apolipoprotein A-II (APOA2)	SPELQAEAK	−1.199	0.000145	0.012035	−1.06	0.288
Ig gamma-3 chain C region (IGHG3)	NQVSLTCLVK	1.162	0.00015	0.01245	1.240	0.00564
Apolipoprotein A-IV (APOA4)	ALVQQMEQLR	−1.191	0.000219	0.018177	−1.229	0.0153
Antithrombin-III (ANT3)	LPGIVAEGR	−1.182	0.000235	0.019505	−1.206	0.0157
Phosphoglycerate kinase 1 (PGK1)	AGGFLMK	−1.127	0.000312	0.025896	−1.074	0.179
Alpha-1-antichymotrypsin (AACT)	EIGELYLPK	−1.162	0.000473	0.039259	−1.218	0.0116
Inter-alpha-trypsin inhibitor heavy chain H2 (ITIH2)	FYNQVSTPLLR	−1.128	0.000489	0.040587	−1.090	0.141

*P*-values were corrected for multiple testing using the false discovery rate. The selected covariates are listed in Supplementary Table 5. ^a^The direction of the fold-change in psychosis patients was not consistent with that in control subjects and so a one-sided test was not conducted (two-sided *P* = 0.00116)

The lasso urbanicity prediction model, fitted to the controls, consisted of one covariate and 13 proteins (13 peptides; Supplementary Table 6b) and had an excellent predictive performance (AUC = 0.90; Supplementary Fig. 2). We attempted to validate the fitted model in the psychosis patients and found that the excellent predictive performance was maintained (AUC = 0.89; Supplementary Fig. 2).

The common functional pathways of the differentially expressed peptides listed in Tables 1 and 2 are summarised in Table 4.

**Table 4:** Common function pathways for the differentially expressed proteins in Tables 1 and 2

Uniprot ID	Abbreviation	Protein ID	Blood coagulation	Cellular energy metabolism	Complement activation	Metabolic process	Acute phase/ inflammatory response	Metal/ion binding	Lipid binding/ transport	Scz reference
P08697	A2AP	Alpha-2-antiplasmin	+				+			^44^
P01011	AACT	Alpha-1-antichymotrypsin				+	+			^45,46^
P02768	ALBU	Serum albumin					+	+	+	^47–49^
P04075	ALDOA	Fructose-bisphosphate aldolase A		+		+		+		^50^
P02760	AMBP	Protein AMBP						+		^51^
P01008	ANT3	Antithrombin-III	+				+	+		^29,44^
P02647	APOA1	Apolipoprotein A-I				+			+	^44,52,53^
P02652	APOA2	Apolipoprotein A-II				+	+		+	^44,54^
P06727	APOA4	Apolipoprotein A-IV				+	+		+	^44,53–55^
P02654	APOC1	Apolipoprotein C-I				+			+	^44,54^
P02656	APOC3	Apolipoprotein C-III				+			+	^44^
P05090	APOD	Apolipoprotein D				+			+	^44,54,56,57^
P02649	APOE	Apolipoprotein E				+			+	^44,58^
P02749	APOH	Beta-2-glycoprotein 1	+			+			+	^12^
O95445	APOM	Apolipoprotein M				+			+	
P04003	C4BPA	C4b-binding protein alpha chain			+	+	+			^59^
P00450	CERU	Ceruloplasmin					+	+		^39,60,61^
P10909	CLUS	Clusterin			+		+			^62–64^
P0C0L4	CO4A	Complement C4-A			+		+			^31^
P02748	CO9	Complement component C9			+					^65^
O75636	FCN3	Ficolin-3			+			+		^65^
P62805	H4	Histone H4				+				
P02790	HEMO	Hemopexin				+	+	+		^55^
P00738	HPT	Haptoglobin				+	+			^12,53,55,60,66–69^
P05155	IC1	Plasma protease C1 inhibitor	+		+		+			
P01860	IGHG3	Ig gamma-3 chain C region		+	+					^70^
P19823	ITIH2	Inter-alpha-trypsin inhibitor heavy chain H2				+				^71^
Q14624	ITIH4	Inter-alpha-trypsin inhibitor heavy chain H4				+	+			^64,72^
P03952	KLKB1	Plasma kallikrein	+			+	+			^44^
P01042	KNG1	Kininogen-1	+				+			
Q99497	PARK7	Protein deglycase DJ-1				+	+			
P00558	PGK1	Phosphoglycerate kinase 1				+				^65,73^
P00491	PNPH	Purine nucleoside phosphorylase				+	+			^74^
P06703	S10A6	Protein S100-A6							+	^75^
P02743	SAMP	Serum amyloid P-component				+	+	+		^52^
P68366	TBA4A	Tubulin alpha-4A chain				+				^76^
P00734	THRB	Prothrombin	+			+	+		+	
P02787	TRFE	Serotransferrin	+			+		+		
P02766	TTHY	Transthyretin				+				^53^
P25311	ZA2G	Zinc-alpha-2-glycoprotein				+	+			^77–83^

## Discussion

We have previously demonstrated that we can successfully detect and reproducibly monitor tens of proteins isolated from serum and DBS^4^ samples using MRM, and here, we demonstrate that we can also do this in stored NBS samples collected between 1975 and 1985 (median CV 10.8%; Supplementary Fig. 2). This has important research implications for countries that routinely store NBS samples and have an associated patient registry (such as Sweden and other Nordic countries) because of the potential to identify prognostic markers for conditions with an onset during early childhood, such as autism, attention deficit hyperactivity disorder and certain types of epilepsy.

We identified serum proteins that differ between first-onset drug-naive patients and controls (Table 2a), 13 of which have previously been associated with schizophrenia (Table 4). We also tested whether any of these proteins were significantly different in NBS samples from newborn babies who later developed schizophrenia and those without a psychiatric diagnosis. The levels of A2AP, CO4A and ANT3 were found to be significantly different at birth (Table 2b). Both A2AP and ANT3 are protease inhibitors, regulating a wide variety of biological processes including coagulation and inflammation and are involved in oxidative stress responses^28–30^.

Genetic variants associated with greater expression of CO4A, a split product of C4, have previously been associated with an increased risk of schizophrenia^31^. The classic complement cascade, of which C4 is a member, is critically involved in synaptic pruning processes^32^. In the immune system, C4 promotes the activation of complement component C3, which in turn modulates inflammation processes in blood. Interestingly, studies in mice indicate that C4 can also mediate synapse elimination during postnatal development^31–33^. In support of our current findings, Hakobyan et al.^34^ previously reported elevated C4 activity in serum from individuals diagnosed with schizophrenia as compared to controls. Futhermore, the volcano plot (Fig. 1b) of the neonatal study results suggest a greater number of proteins with increased levels in the blood of newborn babies who were later diagnosed with schizophrenia than would be expected by chance alone; suggesting an increase in several inflammation-related proteins, as previously reported for adult first-onset schizophrenia patients^12^ and evident in the Cologne study (Fig. 1a).

Although the pathogenesis of schizophrenia remains unknown, increasing evidence from genomic, transcriptomic and proteomic studies supports a role for coagulation, metabolism and inflammation^35–39^. Other predisposing factors include ethnicity, lifestyle, pre-natal and neonatal infections, maternal malnutrition and complications during birth. A common pathological pathway for these predisposing factors could be their common propensity to induce cellular metabolic stress which increase the possibility of oxidative stress and damage^40^. Our findings could suggest that an increased oxidative stress response may represent an inherent schizophrenia vulnerability.

As birth in an urban environment has been associated with an increased risk for psychiatric disorders^10^, we tested whether protein abundance at birth was associated with urbanicity. We found 24 proteins significantly associated with urbanicity in 397 controls (214 urban and 183 rural; Table 3) and confirmed 15 of the 24 proteins in a validation cohort of 97 psychosis patients (45 urban and 52 rural; Table 3). The majority of these 15 differentially expressed proteins relate to immune, especially the acute phase response, and metabolic function (Table 4). The protein with the greatest fold-change was albumin, 3.2-fold and 4.1-fold in controls and psychosis patients, respectively. This is of interest, as albumin has been shown to be the main plasma protein in newborn babies which is modified by oxidative stress, especially through non-bound plasma metals such as iron^41^. Furthermore, several calcium and copper binding proteins were found to differ between urban and rural birth environments, notably ceruloplasmin (CERU; Tables 2 and 3). CERU is a major copper binding protein in plasma^42^ and has previously been associated with neuropsychiatric diseases including schizophrenia^43–47^. Interestingly, altered levels of CERU has also been linked to Wilson’s disease which can present with schizophrenia-like psychosis and can result in misdiagnosis^48,49^. An urban environment is not only associated with more stress and trauma, adverse lifestyles such as drug and alcohol problems among pregnant mothers, but also with air pollution^50^. Previous studies indicate that air pollution in urban locations can affect cognitive and brain development directly. Some air pollutants, such as lead, can cross the blood brain barrier resulting in immune dysregulation and oxidative stress responses at both systemic and brain levels^50^. The predictive performance of the lasso derived model for birth environment was excellent (AUC = 0.90; Supplementary Fig. 2), and this was maintained when we applied the fitted model to the psychosis patient group (AUC = 0.89; Supplementary Fig. 2). Although the ability to distinguish between birth in urban and rural environments per se may not be clinically relevant, it is of great interest that oxidative stress-related protein changes could be identified in both newborn babies who later develop schizophrenia as well as in babies born in an urban setting. The newborn infant is very susceptible to oxidative damage and a wide variety of consumer products and industrial pollutants have been associated with neurotoxicity in distinct developmental time windows^51^. Antioxidant protection for pregnant mothers and newborn infants in the form of dietary supplementation could be evaluated in future epidemiological studies.

There are several limitations to the present study. First, the number of patients and controls from Cologne limit the statistical power of the analysis. Second, given the time lag between birth and schizophrenia diagnosis, relatively few differences in protein abundance were observed. The investigation of a larger number of individuals who later develop schizophrenia will be required to provide the statistical power to identify robust protein abundance differences at birth. Third, as none of the analysed studies have been genotyped, we cannot test whether genetic variants are also associated with elevated levels of CO4A abundance. Fourth, as population density is based on municipality, and some municipalities are geographically large and include smaller and densely populated areas, the indicator of urbanicity used here is crude.

In conclusion, we have demonstrated that reproducible multiplexed quantitation of proteins in stored NBS samples can be achieved using MRM. We have provided further evidence that A2AP, CO4A and ANT3 may be associated with schizophrenia risk and the early disease process. The CO4A association is of particular interest given that genetic variants in CO4A have previously been associated with schizophrenia risk and offer additional support for its potential role in the aetiology of schizophrenia. In addition, we found and validated proteomic differences associated with birth environment. Future biomarker studies based on stored NBS samples used in conjunction with MRM could have the potential to identify risk factors and/or early disease indicators for conditions with onset during early childhood.

## Electronic supplementary material

Supplementary Material
