# Supplementary material for: Schizophrenia-risk and urban birth are associated with proteomic changes in neonatal dried blood spots
Source: Transl Psychiatry. 2017 Dec 18;7:1290. doi: 10.1038/s41398-017-0027-0 (PMC5802534; doi:10.1038/s41398-017-0027-0)
Supplement: Supplementary file 1 — Supplementary Material [file 41398_2017_27_MOESM1_ESM.doc]

Supplementary Information

1. Supplementary methods

1.1 NBS and serum protein sample preparation

For the Cologne samples, 5ul aliquots of serum samples were added to 50mM ammonium bicarbonate followed by disulphide bond reduction and cysteine alkylation using 32.5mM Dithiothreitol (DTT) and 75mM Iodoacetamide (IAA), respectively, and digested overnight with 1:50 (w/w) trypsin. For the neonatal samples, 3mm NBS discs were extracted by re-suspending the discs in 50mM ammonium bicarbonate, followed by disulphide bond reduction and cysteine alkylation using 18.3mM DTT and 46.6mM IAA, respectively, and digested overnight with 1:20 (w/w) trypsin. Digested samples were purified to achieve peptide enrichment using C-18 microfilter 96-well plates. Eluted peptides were dried under vacuum and re-suspended in 50ul of 0.1% formic acid. Isotopically labelled internal standard peptides were spiked into both NBS and serum samples prior to MS run.

The Cologne samples were randomised to allocate equal numbers of patients and controls, and males and females to two plates. Stratified randomisation was also used for the neonatal samples to allocate equal numbers of patients and controls to ten plates. The researchers conducting the sample preparation and MS analysis were blinded to the disease status of the samples.

1.2 Quality control samples

Several quality control (QC) samples were used in this study to monitor method performance and instrument stability. We used commercial serum (Human Sera S7023, Sigma Aldrich) as a quality control (QC) sample, which was aliquoted, digested and run separately to monitor sample preparation variation. Separate QC samples were prepared for NBS and serum studies by pooling equal volumes of digested samples randomly assigned to plate 1. A corresponding pooled sample (NBS or serum) was then aliquoted and run once every day alongside the clinical samples during the total experimental run.

1.3 Statistical quality control

We selected the most abundant peptide-transitions with 80% consistency across MS runs between the endogenous and internal standard, and for the most abundant peptide-transitions with less consistency, we visually checked the peptide for interference from the matrix and manually selected the most abundant transition based on the pooled plate samples. In addition, we applied a relative abundance ratio filter of 1:10 to 10:1, importantly, calculated on the original scale of measurement (for more details see ^1^). After abundance ratio filter exclusions, 68 proteins (128 peptides) and 85 proteins (125 peptides) were available for analysis in the Cologne (serum) and neonatal (NBS) studies respectively (see Supplementary Table 4).

We used principal component analysis (PCA) to identify any outliers based on their relative peptide abundance. Two controls from Cologne were identified as outliers and excluded; the remaining 77 controls were analysed. One northern Swedish control sample was excluded from the neonatal study because of their outlying relative peptide abundances, leaving 644 controls to be analysed (Supplementary Figure 3).

Normalisation

We performed normalisation based on the internal standard to minimize non-biological, systematic variation (technical variation) across MS runs. We used the peak area ratio to normalise the data, that is, the abundance ratio of the endogenous peptide-transitions to their internal standard.

Variance stabilisation

As the variance of biological measurements often increases with intensity, we applied a log_2_ transformation, which is commonly used as a variance stabilising transformation as the variation of the logged abundances is less dependent on the absolute magnitude; skewed distributions become more symmetric and the influence of high-abundance transitions when analysed is reduced ^2^.

1.4 Lasso Regression

Lasso is a penalized method for restricting the residual sum of squares (deviance) and constraining the sum of the absolute values of the coefficients:
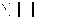
, where *t* is the 'tuning’ parameter. As
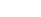
, *t* has no effect and the solutions are the least squares estimates for the full model. For smaller *t* values, solutions are shrunken versions of the least squares estimates with many coefficients decreased to the null value. *t* was defined using ten-fold cross-validation, as the value of *t* minimizing the *t*-penalized residual sum of squares, which is equivalent to maximizing the *t*-penalized log likelihood ^3^. Although the coefficient estimates are biased to be small, a lasso estimator can have smaller error than a standard maximum likelihood estimator when applied to new data. As the analytes were selected based on minimizing the *t*-penalized residual sum of squares, *P*-values for each of the selected analytes are not relevant and not reported.

2.0 Supplementary tables and figures

Supplementary Table 1. Swedish neonatal study patients and controls by year of birth

| Year of birth | Stockholm | | | | | | Västerbotten and Norrbotten | | | | | |
| --- | --- | --- | --- | --- | --- | --- | --- | --- | --- | --- | --- | --- |
|  | Controls | | Psychosis | | Schizophrenia | | Controls | | Psychosis | | Schizophrenia | |
|  | Males | Females | Males | Females | Males | Females | Males | Females | Males | Females | Males | Females |
| 1975-1977 | 71 | 68 | 14 | 14 | 7 | 5 | 40 | 31 | 8 | 16 | 8 | 3 |
| 1978-1980 | 93 | 75 | 22 | 17 | 12 | 7 | 24 | 35 | 5 | 9 | 12 | 5 |
| 1981-1985 | 75 | 80 | 28 | 25 | 5 | 1 | 28 | 24 | 6 | 8 | 5 | 5 |

Supplementary Table 2. Neonatal study missing covariate data

| Covariate | Percent missing (891 participants) |
| --- | --- |
| Sex | 0.0% |
| Year of birth | 0.0% |
| Whether the mother was born abroad | 0.1% |
| Apgar score at 1 minute | 0.9% |
| Apgar score at 5 minutes | 0.7% |
| Parity birth order | 0.0% |
| Whether the child was the first born | 0.0% |
| Caesarean section | 0.0% |
| Completed weeks of gestation | 1.1% |
| Birth weight | 0.3% |
| Length at birth | 0.4% |
| Head circumference | 0.8% |
| Whether the baby was small for their gestation age | 3.3% |
| Age of mother | 0.0% |
| Eclampsia – whether the mother suffered eclampsia | 0.0% |
| Population density per km^2^ (based upon 1991 census data) | 0.0% |

Supplementary Table 3. A summary of the protein peptides available for analysis. Cologne study 68 proteins (128 peptides) and Neonatal study 85 proteins (125 peptides) analysed. 1 = presence; and, 0 = absence.

| Uniprot | Protein | Peptide | Cologne | Neonatal |
| --- | --- | --- | --- | --- |
| P01009 | A1AT | SPLFMGK | 1 | 1 |
| P01009 | A1AT | SVLGQLGITK | 1 | 1 |
| P04217 | A1BG | ATWSGAVLAGR | 1 | 1 |
| P04217 | A1BG | CLAPLEGAR | 1 | 1 |
| P04217 | A1BG | SGLSTGWTQLSK | 1 | 1 |
| P08697 | A2AP | DFLQSLK | 1 | 1 |
| P08697 | A2AP | DSFHLDEQFTVPVEMMQAR | 1 | 0 |
| P08697 | A2AP | FDPSLTQR | 1 | 1 |
| P08697 | A2AP | LCQDLGPGAFR | 0 | 1 |
| P01023 | A2MG | AIGYLNTGYQR | 1 | 1 |
| P01023 | A2MG | NEDSLVFVQTDK | 1 | 1 |
| P01011 | AACT | ADLSGITGAR | 1 | 0 |
| P01011 | AACT | EIGELYLPK | 1 | 1 |
| P01011 | AACT | EQLSLLDR | 1 | 1 |
| P02768 | ALBU | ETYGEMADCCAK | 0 | 1 |
| P04075 | ALDOA | ALANSLACQGK | 0 | 1 |
| P04075 | ALDOA | QLLLTADDR | 0 | 1 |
| P02760 | AMBP | ETLLQDFR | 1 | 1 |
| P02760 | AMBP | TVAACNLPIVR | 1 | 1 |
| P01019 | ANGT | ALQDQLVLVAAK | 1 | 1 |
| P01019 | ANGT | FMQAVTGWK | 1 | 0 |
| P01019 | ANGT | SLDFTELDVAAEK | 1 | 1 |
| P01008 | ANT3 | FDTISEK | 1 | 0 |
| P01008 | ANT3 | LPGIVAEGR | 1 | 1 |
| P02647 | APOA1 | ATEHLSTLSEK | 0 | 1 |
| P02652 | APOA2 | SPELQAEAK | 1 | 1 |
| P06727 | APOA4 | ALVQQMEQLR | 1 | 1 |
| P06727 | APOA4 | IDQNVEELK | 1 | 1 |
| P06727 | APOA4 | ISASAEELR | 1 | 1 |
| P02654 | APOC1 | EFGNTLEDK | 1 | 1 |
| P02654 | APOC1 | EWFSETFQK | 1 | 1 |
| P02655 | APOC2 | ESLSSYWESAK | 1 | 0 |
| P02655 | APOC2 | TAAQNLYEK | 1 | 1 |
| P02655 | APOC2 | TYLPAVDEK | 0 | 1 |
| P02656 | APOC3 | DALSSVQESQVAQQAR | 1 | 1 |
| P02656 | APOC3 | GWVTDGFSSLK | 1 | 1 |
| P55056 | APOC4 | AWFLESK | 1 | 0 |
| P05090 | APOD | VLNQELR | 1 | 1 |
| P02649 | APOE | AATVGSLAGQPLQER | 1 | 1 |
| P02649 | APOE | ALMDETMK | 1 | 0 |
| P02649 | APOE | LEEQAQQIR | 1 | 0 |
| P02649 | APOE | LGPLVEQGR | 1 | 0 |
| P02649 | APOE | SELEEQLTPVAEETR | 1 | 1 |
| P02749 | APOH | EHSSLAFWK | 1 | 1 |
| P02749 | APOH | VSFFCK | 1 | 1 |
| O14791 | APOL1 | LNILNNNYK | 1 | 1 |
| O14791 | APOL1 | VNEPSILEMSR | 1 | 0 |
| O14791 | APOL1 | VTEPISAESGEQVER | 1 | 0 |
| O95445 | APOM | AFLLTPR | 1 | 1 |
| O95445 | APOM | SLTSCLDSK | 1 | 1 |
| P02730 | B3AT | NVELQCLDADDAK | 0 | 1 |
| P30043 | BLVRB | LQAVTDDHIR | 0 | 1 |
| P30043 | BLVRB | NDLSPTTVMSEGAR | 0 | 1 |
| P02747 | C1QC | FNAVLTNPQGDYDTSTGK | 0 | 1 |
| P02747 | C1QC | TNQVNSGGVLLR | 1 | 1 |
| P00736 | C1R | YTTEIIK | 1 | 1 |
| Q9NZP8 | C1RL | GSEAINAPGDNPAK | 1 | 0 |
| P09871 | C1S | LLEVPEGR | 1 | 1 |
| P09871 | C1S | TNFDNDIALVR | 1 | 1 |
| P04003 | C4BPA | EDVYVVGTVLR | 1 | 0 |
| P04003 | C4BPA | FSAICQGDGTWSPR | 1 | 0 |
| P04003 | C4BPA | YTCLPGYVR | 1 | 1 |
| P00915 | CAH1 | ADGLAVIGVLMK | 1 | 0 |
| P00915 | CAH1 | GGPFSDSYR | 0 | 1 |
| P00915 | CAH1 | YSSLAEAASK | 0 | 1 |
| P00918 | CAH2 | GGPLDGTYR | 0 | 1 |
| P00918 | CAH2 | SADFTNFDPR | 0 | 1 |
| P00918 | CAH2 | VVDVLDSIK | 0 | 1 |
| P04040 | CATA | LNVITVGPR | 0 | 1 |
| P04040 | CATA | LSQEDPDYGIR | 0 | 1 |
| P08185 | CBG | GTWTQPFDLASTR | 1 | 0 |
| P08185 | CBG | ITQDAQLK | 1 | 0 |
| Q96IY4 | CBPB2 | DTGTYGFLLPER | 1 | 0 |
| Q96IY4 | CBPB2 | YPLYVLK | 1 | 0 |
| O43866 | CD5L | EATLQDCPSGPWGK | 1 | 0 |
| P00450 | CERU | EVGPTNADPVCLAK | 1 | 1 |
| P00450 | CERU | NNEGTYYSPNYNPQSR | 1 | 0 |
| P00751 | CFAB | DISEVVTPR | 1 | 1 |
| P00751 | CFAB | DLLYIGK | 1 | 1 |
| P00751 | CFAB | EELLPAQDIK | 1 | 1 |
| P00751 | CFAB | YGLVTYATYPK | 1 | 0 |
| P08603 | CFAH | CFEGFGIDGPAIAK | 1 | 0 |
| P10909 | CLUS | FMETVAEK | 1 | 1 |
| P10909 | CLUS | IDSLLENDR | 1 | 1 |
| P06681 | CO2 | AVISPGFDVFAK | 0 | 1 |
| P06681 | CO2 | HAIILLTDGK | 1 | 0 |
| P01024 | CO3 | AGDFLEANYMNLQR | 1 | 0 |
| P01024 | CO3 | VYAYYNLEESCTR | 1 | 0 |
| P0C0L4 | CO4A | DFALLSLQVPLK | 1 | 0 |
| P0C0L4 | CO4A | ITQVLHFTK | 1 | 1 |
| P0C0L4 | CO4A | VLSLAQEQVGGSPEK | 1 | 1 |
| P01031 | CO5 | FQNSAILTIQPK | 0 | 1 |
| P13671 | CO6 | SEYGAALAWEK | 1 | 0 |
| P13671 | CO6 | TLNICEVGTIR | 1 | 0 |
| P07357 | CO8A | MESLGITSR | 1 | 0 |
| P02748 | CO9 | LSPIYNLVPVK | 1 | 1 |
| P02748 | CO9 | VVEESELAR | 1 | 0 |
| P00748 | FA12 | CFEPQLLR | 1 | 0 |
| P00748 | FA12 | VVGGLVALR | 1 | 0 |
| O75636 | FCN3 | YGIDWASGR | 1 | 0 |
| P02765 | FETUA | FSVVYAK | 1 | 1 |
| P02765 | FETUA | HTLNQIDEVK | 1 | 1 |
| P02675 | FIBB | AHYGGFTVQNEANK | 0 | 1 |
| P02751 | FINC | YSFCTDHTVLVQTR | 1 | 0 |
| P50395 | GDIB | DLGTESQIFISR | 0 | 1 |
| P50395 | GDIB | FVSISDLLVPK | 0 | 1 |
| P06396 | GELS | AGALNSNDAFVLK | 1 | 0 |
| P06396 | GELS | SEDCFILDHGK | 1 | 1 |
| P62805 | H4 | DAVTYTEHAK | 0 | 1 |
| P69905 | HBA | FLASVSTVLTSK | 1 | 0 |
| P69905 | HBA | MFLSFPTTK | 1 | 0 |
| P02100 | HBE | LSELHCDK | 0 | 1 |
| P13716 | HEM2 | FASCFYGPFR | 0 | 1 |
| P02790 | HEMO | NFPSPVDAAFR | 1 | 1 |
| P02790 | HEMO | VDGALCMEK | 1 | 1 |
| P05546 | HEP2 | FAFNLYR | 1 | 1 |
| P05546 | HEP2 | IAIDLFK | 1 | 1 |
| P49773 | HINT1 | IIFEDDR | 0 | 1 |
| P00738 | HPT | DYAEVGR | 1 | 0 |
| P00738 | HPT | VGYVSGWGR | 1 | 0 |
| P00738 | HPT | VTSIQDWVQK | 1 | 0 |
| P05155 | IC1 | FQPTLLTLPR | 1 | 1 |
| P05155 | IC1 | TNLESILSYPK | 1 | 0 |
| P01877 | IGHA2 | DASGATFTWTPSSGK | 1 | 0 |
| P01877 | IGHG2 | TTPPMLDSDGSFFLYSK | 1 | 0 |
| P01860 | IGHG3 | DTLMISR | 1 | 1 |
| P01860 | IGHG3 | NQVSLTCLVK | 1 | 1 |
| P01871 | IGHM | QIQVSWLR | 1 | 0 |
| P01871 | IGHM | YAATSQVLLPSK | 1 | 1 |
| P19827 | ITIH1 | GSLVQASEANLQAAQDFVR | 1 | 0 |
| P19827 | ITIH1 | LDAQASFLPK | 1 | 1 |
| P19823 | ITIH2 | FYNQVSTPLLR | 1 | 1 |
| P19823 | ITIH2 | IQPSGGTNINEALLR | 1 | 1 |
| Q14624 | ITIH4 | ETLFSVMPGLK | 1 | 0 |
| Q14624 | ITIH4 | GPDVLTATVSGK | 1 | 1 |
| P00568 | KAD1 | IIFVVGGPGSGK | 0 | 1 |
| P03952 | KLKB1 | LSMDGSPTR | 1 | 1 |
| P01042 | KNG1 | DFVQPPTK | 1 | 1 |
| P01042 | KNG1 | DIPTNSPELEETLTHTITK | 0 | 1 |
| P51884 | LUM | SLEDLQLTHNK | 1 | 1 |
| P15531 | NDKA | DRPFFAGLVK | 0 | 1 |
| Q99497 | PARK7 | DGLILTSR | 0 | 1 |
| P36955 | PEDF | DTDTGALLFIGK | 1 | 0 |
| P36955 | PEDF | ELLDTVTAPQK | 1 | 0 |
| P36955 | PEDF | LQSLFDSPDFSK | 1 | 1 |
| P36955 | PEDF | TVQAVLTVPK | 1 | 1 |
| P05164 | PERM | VVLEGGIDPILR | 0 | 1 |
| P00558 | PGK1 | AGGFLMK | 0 | 1 |
| Q96PD5 | PGRP2 | GCPDVQASLPDAK | 1 | 0 |
| Q96PD5 | PGRP2 | TFTLLDPK | 1 | 1 |
| P80108 | PHLD | NQVVIAAGR | 1 | 0 |
| P00747 | PLMN | FVTWIEGVMR | 1 | 0 |
| P00491 | PNPH | VFGFSLITNK | 0 | 1 |
| P62937 | PPIA | FEDENFILK | 0 | 1 |
| P30041 | PRDX6 | LSILYPATTGR | 0 | 1 |
| P07737 | PROF1 | TLVLLMGK | 0 | 1 |
| P20742 | PZP | MLQITNTGFEMK | 0 | 1 |
| P62826 | RAN | FNVWDTAGQEK | 0 | 1 |
| P43487 | RANG | FLNAENAQK | 0 | 1 |
| P02753 | RET4 | QEELCLAR | 1 | 0 |
| P06703 | S10A6 | LMEDLDR | 0 | 1 |
| P06703 | S10A6 | LQDAEIAR | 0 | 1 |
| P02743 | SAMP | IVLGQEQDSYGGK | 1 | 1 |
| P04278 | SHBG | IALGGLLFPASNLR | 1 | 1 |
| P31948 | STIP1 | AAALEFLNR | 0 | 1 |
| P31948 | STIP1 | LMDVGLIAIR | 0 | 1 |
| P68366 | TBA4A | EIIDPVLDR | 0 | 1 |
| P05452 | TETN | EQQALQTVCLK | 1 | 0 |
| P05543 | THBG | NALALFVLPK | 0 | 1 |
| P10599 | THIO | VGEFSGANK | 0 | 1 |
| P00734 | THRB | ELLESYIDGR | 1 | 1 |
| P00734 | THRB | SGIECQLWR | 1 | 1 |
| P60174 | TPIS | FFVGGNWK | 0 | 1 |
| P60174 | TPIS | VVLAYEPVWAIGTGK | 0 | 1 |
| P02787 | TRFE | EGYYGYTGAFR | 0 | 1 |
| P02766 | TTHY | AADDTWEPFASGK | 1 | 1 |
| P02766 | TTHY | VLDAVR | 1 | 1 |
| P04004 | VTNC | DWHGVPGQVDAAMAGR | 0 | 1 |
| P25311 | ZA2G | AGEVQEPELR | 0 | 1 |

Supplementary Table 4 (a). Covariates selected for the peptide-transitions listed in Table 2 (a) from the schizophrenia association validation analysis. Note that there were only two covariates, age and sex, available for selection in the patients and controls from Cologne.

| Protein | Peptide | Variable selection in patient-control association analysis | |
| --- | --- | --- | --- |
|  |  | 1 | 2 |
| Haptoglobin (HPT) | VTSIQDWVQK | age | -- |
| Haptoglobin (HPT) | DYAEVGR | age | -- |
| Haptoglobin (HPT) | VGYVSGWGR | age | -- |
| Plasma protease C1 inhibitor (IC1) | TNLESILSYPK | age | sex |
| Apolipoprotein C-III (APOC3) | GWVTDGFSSLK | sex | -- |
| Apolipoprotein A-IV (APOA4) | IDQNVEELK | sex | -- |
| Plasma protease C1 inhibitor (IC1) | FQPTLLTLPR | age | sex |
| Apolipoprotein C-III (APOC3) | DALSSVQESQVAQQAR | -- | -- |
| Antithrombin-III (ANT3) | FDTISEK | age | sex |
| Antithrombin-III (ANT3) | LPGIVAEGR | age | sex |
| Complement C4-A (CO4A) | VLSLAQEQVGGSPEK | -- | -- |
| Alpha-1-antichymotrypsin (AACT) | EQLSLLDR | sex | -- |
| Apolipoprotein A-II (APOA2) | SPELQAEAK | -- | -- |
| Inter-alpha-trypsin inhibitor heavy chain H4 (ITIH4) | GPDVLTATVSGK | age | -- |
| Complement component C9 (CO9) | VVEESELAR | -- | -- |
| Apolipoprotein C-I (APOC1) | EFGNTLEDK | age | -- |
| Complement component C9 (CO9) | LSPIYNLVPVK | -- | -- |
| Complement C4-A (CO4A) | ITQVLHFTK | -- | -- |
| Ficolin-3 (FCN3) | YGIDWASGR | age | sex |
| Apolipoprotein A-IV (APOA4) | ISASAEELR | -- | -- |
| Alpha-2-antiplasmin (A2AP) | FDPSLTQR | age | -- |
| Beta-2-glycoprotein 1 (APOH) | EHSSLAFWK | age | sex |

Supplementary Table 4 (b). Covariates selected for the peptide-transitions listed in Table 2 (c) from the schizophrenia association analysis. Note that we found no overall proteomic differences between the Stockholm and Northern Sweden (Västerbotten and Norrbotten) studies.

| Protein | Peptide | | Variable selection in urban-rural association analysis | | | | | | | | | |
| --- | --- | --- | --- | --- | --- | --- | --- | --- | --- | --- | --- | --- |
|  |  |  | 1 | | 2 | | 3 | | 4 | | 5 | |
| Transthyretin (TTHY) | | VLDAVR | | year of birth (linear) | | sex | | birth weight | | -- | | -- |
| Alpha-2-antiplasmin (A2AP) | | FDPSLTQR | | small for gestation age | | Apgar score at 5 minutes | | -- | | -- | | -- |
| Protein AMBP (AMBP) | | ETLLQDFR | | Apgar score at 5 minutes | | C-section | | year of birth (linear) | | -- | | -- |
| Serotransferrin (TRFE) | | EGYYGYTGAFR | | mother born abroad | | -- | | -- | | -- | | -- |
| C4b-binding protein alpha chain (C4BPA) | | YTCLPGYVR | | completed weeks of gestation | | -- | | -- | | -- | | -- |
| Complement C4-A (CO4A) | | ITQVLHFTK | | birth weight | | year of birth (linear) | | first born | | -- | | -- |
| Tubulin alpha-4A chain (TBA4A) | | EIIDPVLDR | | year of birth (linear) | | small for gestation age | | birth length | | -- | | -- |
| Apolipoprotein A-II (APOA2) | | SPELQAEAK | | sex | | small for gestation age | | C-section | | -- | | -- |
| Clusterin (CLUS) | | IDSLLENDR | | small for gestation age | | Apgar score at 5 minutes | | year of birth (smooth) | | -- | | -- |
| Ig gamma-3 chain C region (IGHG3) | | DTLMISR | | year of birth (linear) | | completed weeks of gestation | | -- | | -- | | -- |
| Kininogen-1 (KNG1) | | DFVQPPTK | | -- | | -- | | -- | | -- | | -- |
| Ig gamma-3 chain C region (IGHG3) | | NQVSLTCLVK | | -- | | -- | | -- | | -- | | -- |
| Apolipoprotein A-IV (APOA4) | | IDQNVEELK | | birth length | | completed weeks of gestation | | year of birth (linear) | | -- | | -- |
| Apolipoprotein D (APOD) | | VLNQELR | | Apgar score at 5 minutes | | -- | | -- | | -- | | -- |
| Purine nucleoside phosphorylase (PNPH) | | VFGFSLITNK | | Mother’s age | | C-section | | -- | | -- | | -- |
| Apolipoprotein A-I (APOA1) | | ATEHLSTLSEK | | year of birth (linear) | | C-section | | -- | | -- | | -- |
| Alpha-2-antiplasmin (A2AP) | | DFLQSLK | | small for gestation age | | year of birth (linear) | | -- | | -- | | -- |
| Antithrombin-III (ANT3) | | LPGIVAEGR | | -- | | -- | | -- | | -- | | -- |
| Histone H4 (H4) | | DAVTYTEHAK | | year of birth (linear) | | sex | | -- | | -- | | -- |

Supplementary Table 5. Covariates selected for the peptide-transitions listed in Table 3 in the urbanicity association analysis in 397 controls from the neonatal study.

| Protein | Peptide | Variable selection in urban-rural association analysis | | | | |
| --- | --- | --- | --- | --- | --- | --- |
|  |  | 1 | 2 | 3 | 4 | 5 |
| Serum albumin (ALBU) | ETYGEMADCCAK | year of birth (linear) | completed weeks of gestation |  |  |  |
| Plasma kallikrein (KLKB1) | LSMDGSPTR | year of birth (linear) | parity birth order | -- | -- | -- |
| Zinc-alpha-2-glycoprotein (ZA2G) | AGEVQEPELR | -- | -- | -- | -- | -- |
| Apolipoprotein A-IV (APOA4) | IDQNVEELK | birth weight | year of birth (smooth) | completed weeks of gestation | -- | -- |
| Apolipoprotein C-I (APOC1) | EFGNTLEDK | year of birth (smooth) | eclampsia | -- | -- | -- |
| Ceruloplasmin (CERU) | EVGPTNADPVCLAK | completed weeks of gestation | -- | -- | -- | -- |
| Hemopexin (HEMO) | VDGALCMEK | year of birth (smooth) | eclampsia | -- | -- | -- |
| Protein deglycase DJ-1 (PARK7) | DGLILTSR | year of birth (smooth) | -- | -- | -- | -- |
| Apolipoprotein M (APOM) | SLTSCLDSK | -- | -- | -- | -- | -- |
| Fructose-bisphosphate aldolase A (ALDOA) | ALANSLACQGK | year of birth (smooth) | -- | -- | -- | -- |
| Clusterin (CLUS) | IDSLLENDR | parity birth order | year of birth (smooth) | -- | -- | -- |
| C4b-binding protein alpha chain (C4BPA) | YTCLPGYVR | completed weeks of gestation | -- | -- | -- | -- |
| Apolipoprotein A-I (APOA1) | ATEHLSTLSEK | year of birth (smooth) | -- | -- | -- | -- |
| Prothrombin (THRB) | SGIECQLWR | -- | -- | -- | -- | -- |
| Apolipoprotein E (APOE) | AATVGSLAGQPLQER | -- | -- | -- | -- | -- |
| Serum amyloid P-component (SAMP) | IVLGQEQDSYGGK | Apgar score at 5 minutes | -- | -- | -- | -- |
| Alpha-1-antichymotrypsin (AACT) | EQLSLLDR | year of birth (smooth) | -- | -- | -- | -- |
| Protein AMBP (AMBP) | TVAACNLPIVR | -- | -- | -- | -- | -- |
| Protein S100-A6 (S10A6) | LQDAEIAR | Mother’s age | -- | -- | -- | -- |
| Apolipoprotein A-II (APOA2) | SPELQAEAK | completed weeks of gestation | -- | -- | -- | -- |
| Ig gamma-3 chain C regionIGHG3 | NQVSLTCLVK | -- | -- | -- | -- | -- |
| Apolipoprotein A-IV (APOA4) | ALVQQMEQLR | birth weight | -- | -- | -- | -- |
| Antithrombin-III (ANT3) | LPGIVAEGR | -- | -- | -- | -- | -- |
| Phosphoglycerate kinase 1 (PGK1) | AGGFLMK | Mother’s age | -- | -- | -- | -- |
| Alpha-1-antichymotrypsin (AACT) | EIGELYLPK | year of birth (smooth) | -- | -- | -- | -- |
| Inter-alpha-trypsin inhibitor heavy chain H2 (ITIH2) | FYNQVSTPLLR | year of birth (smooth) | -- | -- | -- | -- |

Supplementary Table 6 (a). The prediction model found using lasso regression with 10-fold cross-validation applied to the 60 first-onset drug naïve schizophrenia patients and 77 controls from Cologne.

| Predictors | | Lasso regression coefficient |
| --- | --- | --- |
| Protein | Peptide |
| ANT3 | LPGIVAEGR | 0.273 |
| APOA2 | SPELQAEAK | -1.368 |
| APOA4 | IDQNVEELK | -0.532 |
| APOC3 | GWVTDGFSSLK | -0.634 |
| APOH | EHSSLAFWK | 0.157 |
| FCN3 | YGIDWASGR | 0.0270 |
| FETUA | HTLNQIDEVK | -0.608 |
| HPT | VTSIQDWVQK | 0.340 |
| IC1 | TNLESILSYPK | 0.370 |
| ITIH4 | GPDVLTATVSGK | 0.598 |
| THRB | SGIECQLWR | 0.602 |

Supplementary Table 6 (b). Urbanicity prediction model found using lasso regression with 10-fold cross-validation applied to 397 controls from the neonatal study.

| Predictors | | Lasso regression coefficient |
| --- | --- | --- |
| Year of Birth | | 0.148 |
| Protein | Peptide |  |
| ALBU | ETYGEMADCCAK | 0.507 |
| ANT3 | LPGIVAEGR | -0.156 |
| APOA2 | SPELQAEAK | -0.0677 |
| APOC1 | EFGNTLEDK | -0.459 |
| APOE | AATVGSLAGQPLQER | -0.110 |
| C4BPA | YTCLPGYVR | 0.180 |
| CERU | EVGPTNADPVCLAK | 0.103 |
| IGHG3 | NQVSLTCLVK | 0.0310 |
| KLKB1 | LSMDGSPTR | 0.262 |
| PGK1 | AGGFLMK | -0.234 |
| S10A6 | LQDAEIAR | -0.113 |
| THRB | SGIECQLWR | 0.119 |
| ZA2G | AGEVQEPELR | -0.937 |

Supplementary Figure 1 (a). CVs calculated for the pooled plate 1 samples, included on plates 1 (seven sample injections) and 2 (four sample injections) of the Cologne study. The median CV was 7.23% (6.54% in plate 1 and 7.92% in plate2) based upon 68 proteins (128 peptides).


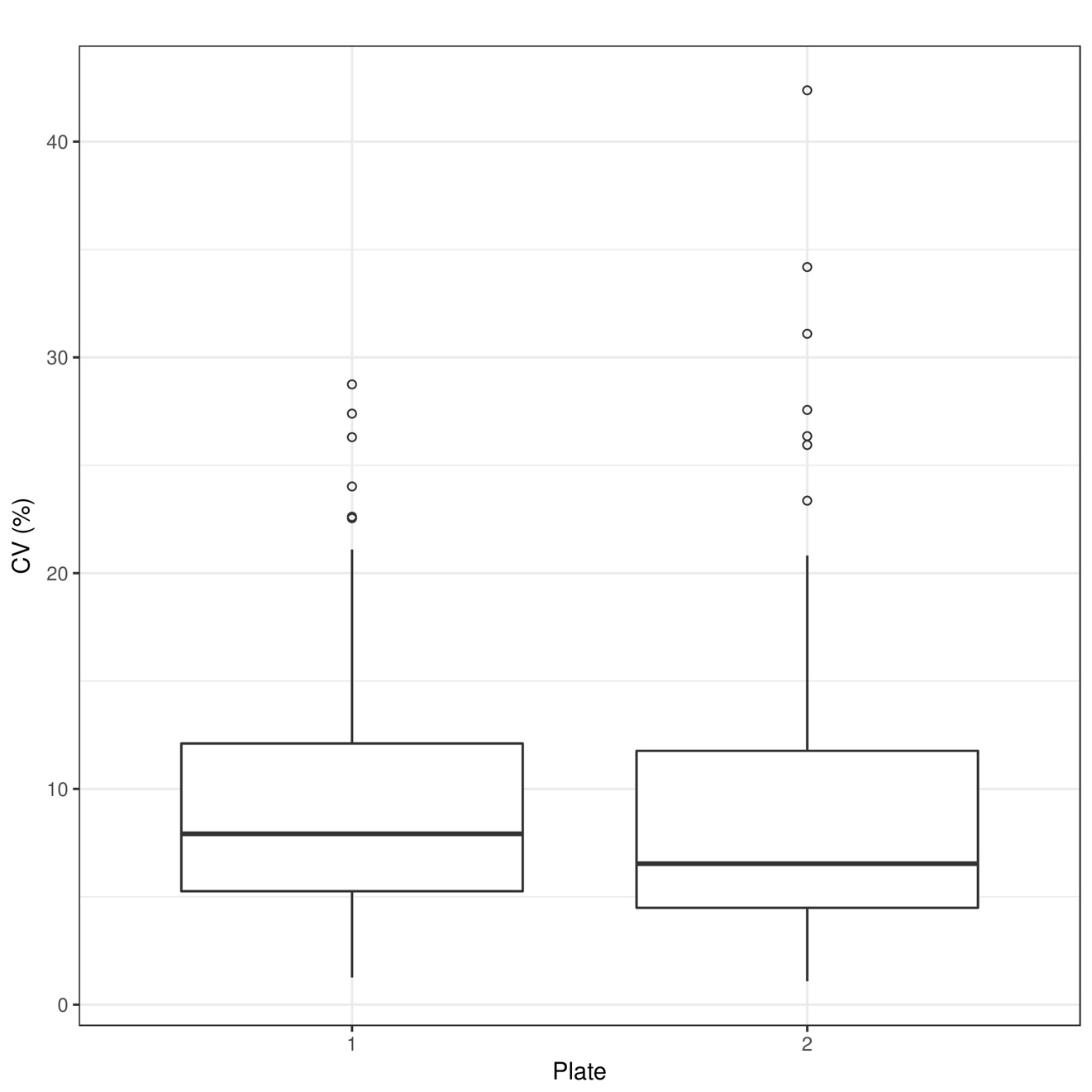


Supplementary Figure 1 (b). CVs calculated for the pooled plate 1 samples, included on plates 2 to 10 (five sample injections) of the Neonatal study. The median CV was 10.83% (range 9.50 to 11.52%) based upon 85 proteins (125 peptides).


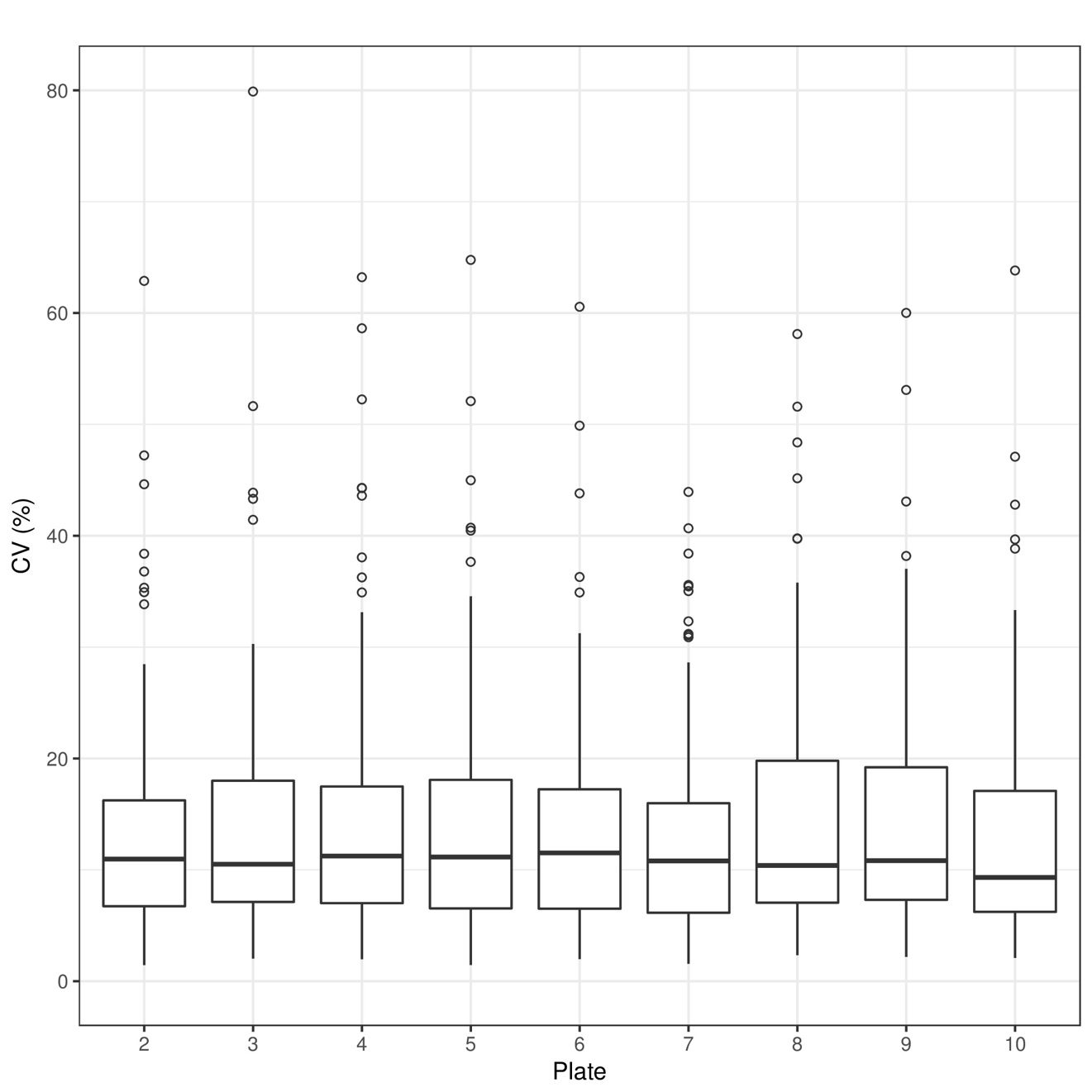


Supplementary Figure 2. Receiver operating characteristic (ROC) curves for urbanicity predictive performance using the 13 predictors selected using lasso regression with 10-fold cross-validation (selected predictors and their coefficients listed in Supplementary Table 6b). Discovery – 397 controls; and, Validation – 97 psychosis patients, sens = sensitivity and spec = specificity.


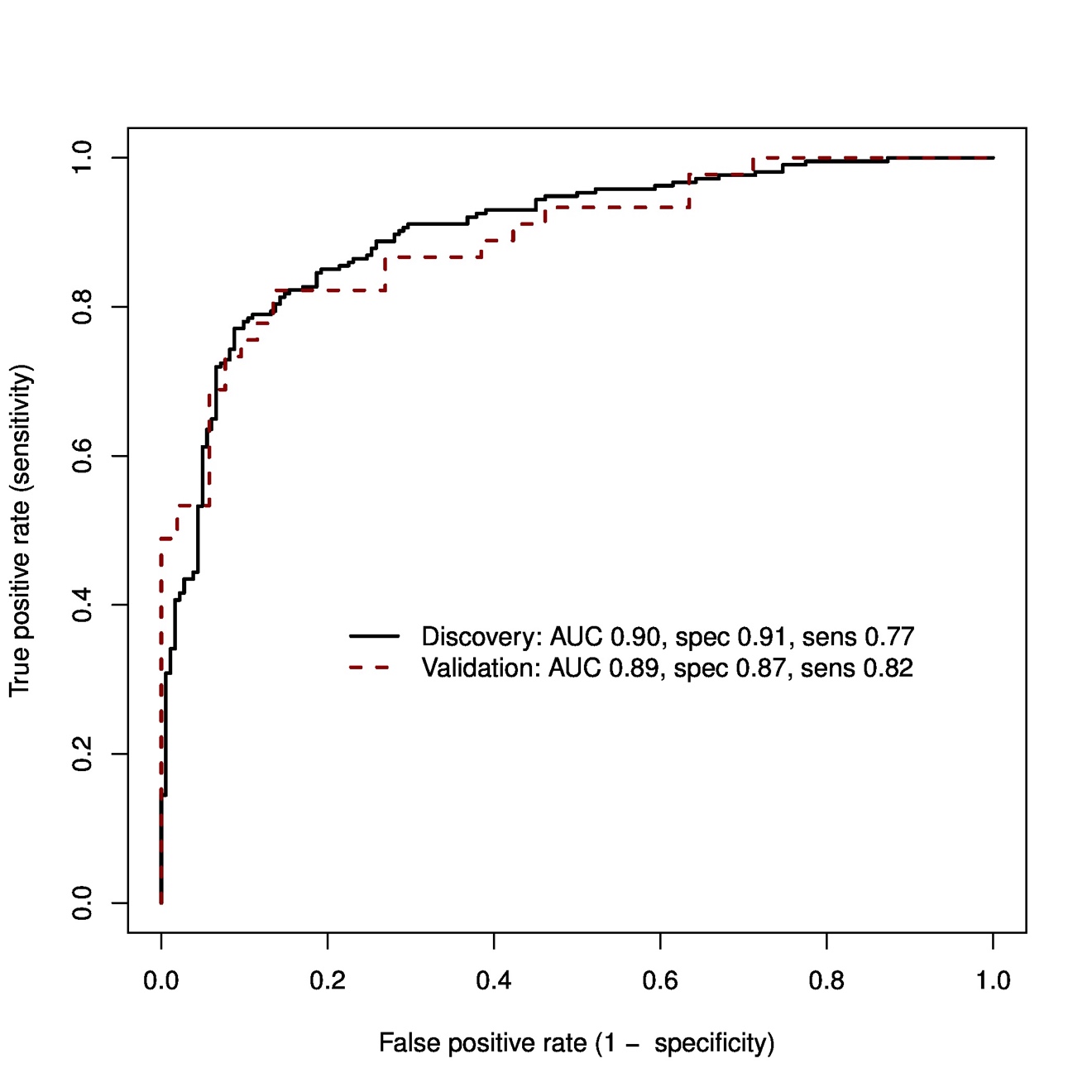


Supplementary Figure 3. Boxplots of relative peptide abundance by run. Northern Sweden, Västerbotten and Norrbotten, control (run 1146) was excluded as an outlier based on their relative protein abundances.


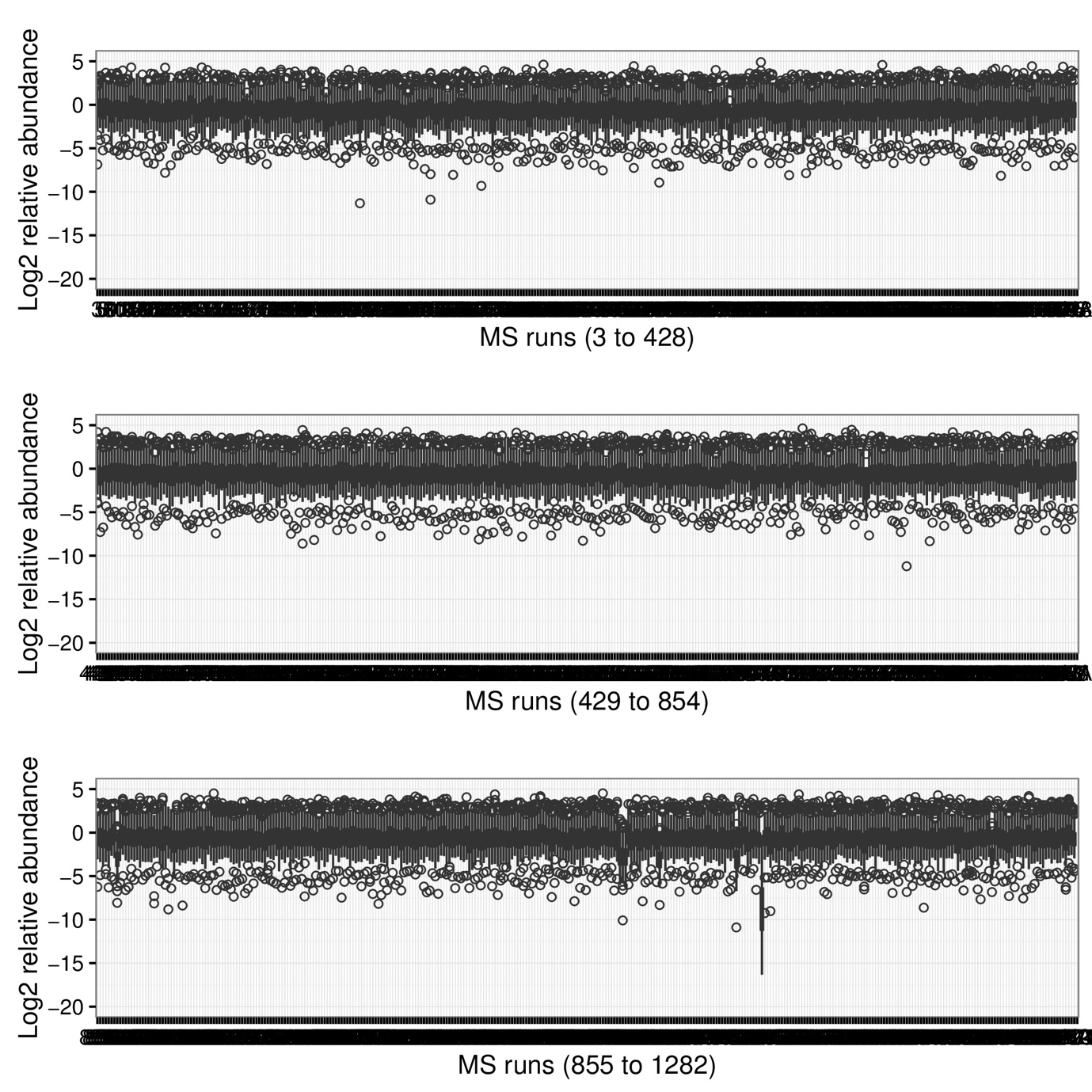


3.0 References

1 Ozcan, S. *et al.* Towards reproducible MRM based biomarker discovery using dried blood spots. *Scientific Reports* (in press).

2 Dudoit, S., Yang, Y. H., Callow, M. J. & Speed, T. P. Statistical methods for identifying differentially expressed genes in replicated cDNA microarray experiments. *Stat Sinica* **12**, 111-139 (2002).

3 Hastie, T., Tibshirani, R. & Friedman, J. *The Elements of Statistical Learning: Data Mining, Inference, and Prediction*. 5th Edition edn, (Springer, New York, NY, USA, 2001).
